# Development of an in-house, one-step RT-qPCR mix and optimized MS2 detection primers for hepatitis A virus and norovirus detection in berries^1^

**DOI:** 10.1016/j.mex.2025.103703

**Published:** 2025-11-01

**Authors:** Hui Zhi Low, Christina Böhnlein, Charles M.A.P. Franz

**Affiliations:** Department of Microbiology and Biotechnology, Max Rubner-Institut, Federal Research Institute of Nutrition and Food, Hermann-Weigmann-Str. 1 24103 Kiel, Germany

**Keywords:** Real-time PCR, virus detection, berries, MS2 bacteriophage, ISO 15216–2

## Abstract

One-step, reverse transcriptase-quantitative PCR (RT-qPCR) is the primary method for detecting foodborne viruses in food matrices. The ISO 15216-2:2019 serves as the international standard for detecting human norovirus GI, GII, and hepatitis A virus. Some food matrices, such as berries, tend to co-purify PCR inhibitors with viral RNA, which can lead to false-negative results. To prevent this, the protocol includes extensive control approaches. However, the high cost of commercial RT-qPCR kits makes large-scale virus testing expensive and inaccessible. To address this, we developed an in-house, one-step RT-qPCR mix using commercial, next-generation enzymes with improved resistance to PCR inhibitors and with enhanced performance. The in-house mix offers a more cost-effective alternative to expensive and outdated commercial mixes. In this paper, we describe:

• the development of an in-house, one-step multiplexable RT-qPCR protocol and optimization process as a reference for laboratories seeking to develop their own in-house protocols.

• altered and optimized, previously described primers for the MS2 virus, further improving the efficiency of its detection and its reliability as a process control virus.

## Graphical abstract

**Figure:**
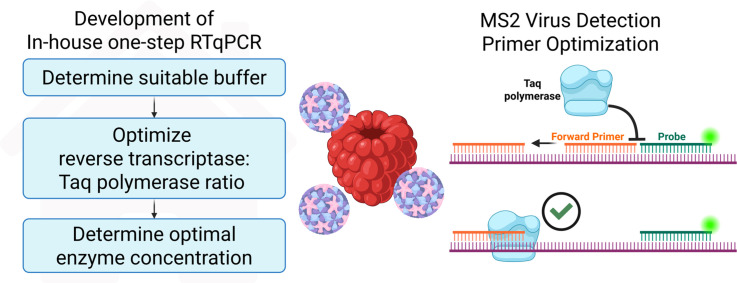

## Specifications table

**Table:** 

**Subject area**	Biochemistry, Genetics and Molecular Biology
**More specific subject area**	Virus detection
**Name of your method**	One-step reverse transcriptase quantitative polymerase chain reaction
**Name and reference of original method**	Dreier, Jens; Störmer, Melanie; Kleesiek, Knut [1]: Use of bacteriophage MS2 as an internal control in viral reverse transcription-PCR assays. In *Journal of clinical microbiology* 43 (9), pp. 4551–4557. DOI: 10.1128/JCM.43.9.4551–4557.2005.
**Resource availability**	All required resources to reproduce this method are described in this article.

## Background

The one-step reverse transcriptase–quantitative polymerase chain reaction (RT-qPCR) remains the primary technique for detecting food-associated viruses within complex food matrices. In particular, the ISO 15216-2:2019 standard [2] serves as the international benchmark for identifying human norovirus genogroups I and II (HuNoV-GI, -GII), as well as hepatitis A virus (HAV) [3,4]. This protocol describes the purification of virus particles from a variety of food and environmental matrices and makes specifications and recommendations relating to RNA purification and molecular detection of the viral RNA using a one-step RT-qPCR.

During virus extraction from food matrices, substances that inhibit RT-qPCR are often co-purified with the viral RNA, necessitating extensive controls to avoid false-negative results [5]. This yields a resource-intensive plate layout—up to 40 wells per sample for singleplex assays (12 for sample, 28 for process control virus (PCV) and controls). Multiplexing cuts this to 18 wells per sample (4 sample, 14 PCV/controls). Commercial one-step RT-qPCR kits from well-known suppliers are very costly, making it exorbitantly expensive to assay for viruses according to ISO 15216-2:2019 at larger scale. Using the Invitrogen RNA UltraSense™ kit mentioned in ISO 15216-2:2019, costs per sample are around 150 € (prices from Germany, April 2025; before VAT) just for the one-step RT-qPCR mix, not including primers and probes. There are provisions to reduce the number of control wells when testing for known matrices with low copurification of RT-qPCR inhibitors, thereby saving costs. However, this is not feasible when analyzing food matrices for which PCR inhibition has not yet been evaluated, as well as for difficult matrices that are prone to contain PCR inhibitors that tend to be copurified with the viral RNA, for example berries.

In order to save costs, we aimed to establish an in-house, one-step RT-qPCR mix. A very commonly used mix is the RNA UltraSense kit that is mentioned in the informative part (Annex C) of the ISO 15216-2:2019 standard, which comes with a very high reaction cost per 25 µL well of 3.69 €. It should be noted that the informative annex of ISO 15216-2:2019 is non-normative: it neither constitutes a recommendation nor mandates the use of the RNA UltraSense kit. However, many investigations published based on this protocol have indeed used this kit [[6], [7], [8], [9], [10], [11]]. Based on a literature survey and manufacturer information, the RNA Ultrasense kit dates back to at least 2006 and uses the SuperScript™ III reverse-transcriptase and the Platinum™ Taq polymerase enzyme. However, updated versions of both the reverse transcriptase and Taq polymerase (Superscript IV and Platinum II Taq) have since been released, which are claimed to have better inhibitor resistance and reactivity according to manufacturer whitepaper and comparison studies [12,13]. This study aimed to use these new-generation enzymes to create a more cost-effective alternative that is also more sensitive than the RNA Ultrasense kit. In the process, one-step RT-qPCR primers for the MS2 process control virus were also improved.

## Method details

***External control RNA (ecRNA).*** ecRNA was generated through *in vitro* transcription of PCR-amplified target sequences, following the guidelines provided in Annex G of ISO 15216-2:2019. T7 promoter regions were introduced upstream of the target sequences, with random nucleotides flanking the promoter-target sequence. These sequences were synthesized via gene synthesis (Eurofins, Ebersberg, Germany) and subsequently amplified using primers flanking the sequences with Q5 High-Fidelity Polymerase (New England Biolabs (NEB), Ipswich, MA, USA). The gene synthesis sequences and amplification primers are provided in Supplementary Table 1. Resulting PCR products were purified using the Monarch PCR Purification Kit (NEB) and transcribed using the HiScribe T7 High Yield RNA Synthesis Kit (NEB). The RNA products were then purified with the Monarch RNA Cleanup Kit, 500 µg (NEB). RNA concentration was measured by the A_260_ method using a NanoDrop ND-1000 (Thermo Fisher Scientific, Waltham, MA, USA). RNA was diluted to 120 ng/µL (HAV), 63 ng/µL (HuNoV-GI) and 65 ng/µL (HuNoV-GII), corresponding to 10^12^ copies per µL, and aliquoted for long-term storage in THE RNA Storage Solution (Thermo Fisher Scientific) at −70 °C.

***Primers.*** Real-time-PCR primers and hydrolysis probes were used in accordance with Annex D of ISO 15216-2:2019 for the detection of HAV, norovirus GI, and norovirus GII. Singleplex and multiplex probes were synthesized according to Table 1 (Metabion, Planegg, Germany). Primers for the detection of the MS2 process control virus were adapted from Dreier et al [1]. An alternative forward primer binding site upstream of the original forward primer was identified with the help of Primer-BLAST [14].

**Table 1:** List of primers, probes and modifications used in virus detection.

Virus	Primers	(5′ - 3′)	Final concentration (nM)	Singleplex Modifications (5′; 3′)	Multiplex Modifications (5′; 3′)	References
HAV	Forward	TCA CCG TTT GCC TAG	500			[15]
	Reverse	GGA GAG CCC TGG AAG AAA G	900			
	Probe	CCT GAA CCT GCA GGA ATT AA	250	FAM; MGBNFQ	FAM; MGBNFQ	
HuNoV-GI	Forward	CGC TGG ATG CGN TTC CAT	500			[16,17]
	Reverse	CCT TAG ACG CCA TCA TTT AC	900			
	Probe	TGG ACA GGA GAY CGC RAT CT	250	FAM; TAMRA	TET; BHQ-1	
HuNoV-GII	Forward	ATG TTC AGR TGG ATG AGR TTC TCW GA	500			[15,18,19]
	Reverse	TCG ACG CCA TCT TCA TTC ACA	900			
	Probe	AGC ACG TGG GAG GGC GAT CG	250	FAM; TAMRA	Texas red; BHQ2	

***One-step RT-qPCR reaction conditions.*** All RT-qPCR reactions were performed in 25 µL reaction volumes and run on the Bio-Rad CFX96 Touch Real-Time PCR Detection System. Cycling conditions were adapted from Annex C of ISO 15216-2:2019. The same reaction conditions were used for commercial kits and for in-house mix with SuperScript IV reverse transcriptase and Platinum II Taq Hot-Start DNA polymerase (Thermo Fisher Scientific). Reverse transcription was carried out at 55 °C for 1 hour, followed by enzyme inactivation and Taq polymerase activation (hot start) at 95 °C for 5 min. This was followed by 45 amplification cycles consisting of denaturation at 95 °C for 15 s, annealing at 60 °C for 1 min, and extension at 65 °C for 1 min. Fluorescence data were collected at the end of the extension step in each cycle.

***Commercial one-step RT-qPCR kits and establishment of an in-house, one-step RT-qPCR.*** Commercial one-step RT-qPCR kits used in this study included the RNA UltraSense One-Step Quantitative RT-PCR System (Thermo Fisher Scientific) and Luna ® Probe One-Step RT-qPCR 4X Mix with UDG (NEB). Commercial kits were used according to manufacturer’s instructions in 25 µL reaction volumes. An in-house, one-step RT-qPCR was established with the addition of 0.4 mM of each dNTP (NEB), 0.5 mM DTT, 800 µg/mL UltraPure™ BSA (both Thermo Fisher Scientific) and 5 U RNase Inhibitor Hu (Qiagen, Hilden, Germany). A total of 4 data points across 2 experiments were used for the statistical analysis with outlier exclusion (see Statistical analysis).

For buffer system optimization, 1 U each of SuperScript™ IV reverse transcriptase and Platinum™ II Taq Hot-Start DNA polymerase, along with 5 U of RNase Inhibitor Hu were used. This was tested with higher (10^6^) and lower (10^2^) copy numbers of norovirus GI ecRNA over two independent experiments, each comprising two technical replicates.

To determine optimal ratio of reverse transcriptase to Taq polymerase, 0.1–1.6 units of SuperScript™ IV reverse transcriptase were combined with 1 unit of Platinum™ II Taq Hot-Start DNA polymerase using higher (10^6^) and lower (10^3^, 10^2^) copy numbers of norovirus GI ecRNA across three independent experiments, each with 2–3 technical replicates. A total of 7 data points across 3 experiments were used for the statistical analysis with outlier exclusion (see Statistical analysis).

***Viral RNA isolation from berries according to ISO 15216-2:2019.*** Frozen raspberries were purchased from local supermarkets. MS2 process control virus was obtained from our own department’s Bacteriophage Competence Center group. For multiplex virus detection, the National Reference Laboratory for Foodborne Viruses at the German Federal Institute for Risk Assessment (BfR) provided strawberry samples that were artificially contaminated with HuNoV-GI, HuNoV-GII and HAV. All centrifugation steps were performed at 10,000 x g, 5 °C.

For each viral RNA purification, 25 g portions of raspberries were thawed and spiked with 10 µL of MS2 process control virus which contained 5.5 × 10^6^ PFU, and transferred to a 400 mL filter bag. MS2-spiked berry samples (with and without additional spiked viruses) were then incubated on a rocking shaker (60 rpm) for 20 min in TGBE buffer with 1900 U pectinase from *Aspergillus aculeatus* (Sigma), determining and adjusting the pH to between 9–10 with 13 M NaOH every 10 min. For each pH adjustment, 10 min was added to the incubation time for up to 3 times. The eluate in the filter chamber was centrifuged for 30 min and the supernatant was neutralized to pH 7 with 10 M HCl. A quarter-volume of 5 x PEG/NaCl solution was added to the supernatant and virus precipitation occurred for 60 min at 5 °C, with constant rocking at 60 rpm. Precipitated virus was pelleted for 35 min and the pellet was resuspended in 500 µL PBS. The virus suspension was vortexed with a 500 µL chloroform/butanol mixture (50/50) and centrifuged for 15 min. The whole aqueous top phase (≈ 500 µL) was removed for RNA isolation with the NucleoSpin Virus Mini Kit (Macherey Nagel, Düren, Germany) according to manufacturer’s instructions, with reagent volumes for the lysis step scaled to the larger input (RAV1 lysis buffer 2 mL and ethanol 2 mL). The sample was applied to the spin column in successive aliquots and the column was reloaded as required until the full volume had passed through. Washing and elution steps (100 µL nuclease-free water) were performed according to the manufacturer’s protocol.

For MS2 recovery, two independent experiments were performed, each using three raspberry samples.

***Multiplex detection of viruses.*** 25 g frozen strawberry samples artificially contaminated with HuNoV-GI.4 (stool origin, virusbank NRL 59-07), HuNoV-GII.P16/GII.4 Sydney 2012 (stool origin, virusbank NRL 008-23) and HAV HM 175 (cell culture origin) were provided by the National Reference Laboratory for Foodborne Viruses from the German Federal Institute for Risk Assessment as part of an interlaboratory comparison study. Sample 1 was contaminated with 3.2 × 10^4^ genome copies of HuNoV-GI.4 and 1.1 × 10^4^ genome copies of HAV HM 175 per 25 g sample. Sample 2 was contaminated with 1.6 × 10^5^ genome copies of HuNoV-GII.P16/GII.4 and 1.1 × 10^4^ genome copies of HAV HM 175 per 25 g sample.

Primer and probe concentrations for each virus were added to the reaction at the same final concentrations as in the corresponding singleplex assays.

***Software tools.*** qPCR results were analyzed using the Bio-Rad CFX Maestro 2.3.

**Fig. 1:**
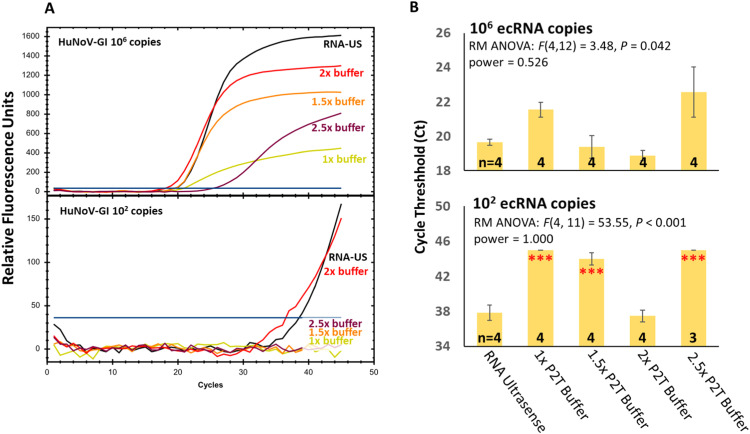
Optimization of one-step RT-qPCR reaction conditions. Various dilutions of Platinum II Taq Hot-Start DNA Polymerase buffer were first optimized relative to the use of 1 U SuperScript IV Reverse Transcriptase and 1 U Platinum II Taq Hot-Start DNA Polymerase per well. Reactions using the RNA UltraSense (RNA-US) kit served as a positive control. (A) Representative amplification plots and (B) mean Ct values with standard error of measurement (SEM) obtained from 2 independent experiments, each with 2 technical replicates. Statistical analysis: repeated-measures one-way ANOVA (RM-ANOVA); n = sample number; ***P < 0.001. Outliers deviating by >4 Ct from the dataset mean were excluded.

**Fig. 2:**
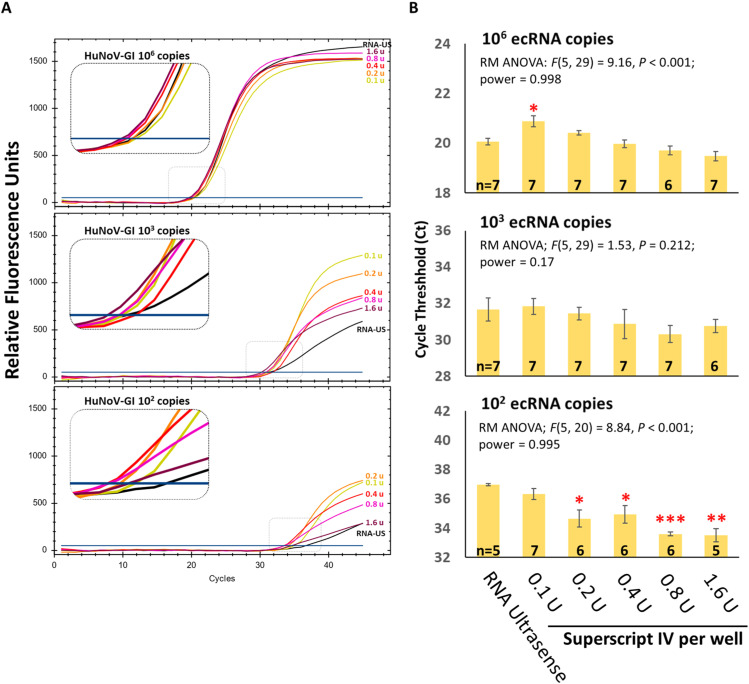
Various concentrations of the reverse transcriptase were tested against 1 U of the Taq polymerase per well. Reactions using the RNA UltraSense (RNA-US) kit served as a positive control. (A) Representative amplification plots and (B) mean Ct values after outlier removal, with SEM from 3 independent experiments, each with 2–3 technical replicates. Statistical analysis: repeated-measures one-way ANOVA (RM-ANOVA); n = sample number; *P < 0.05; **P < 0.01; ***P < 0.001. Outliers deviating by >4 Ct from the dataset mean were excluded.

***Statistical analysis*** was performed using SigmaPlot 14.0. For Fig. 1, Fig. 2, outliers were defined as amplification reactions with Ct values deviating by >4 cycles from the dataset mean, corresponding to >16-fold variation in template quantity, and were excluded prior to statistical analysis to account for potential technical artifacts.

## Method validation

***qPCR Buffer***. RT-qPCR reactions with varying concentrations of the Platinum II Taq PCR buffer showed that a 2× buffer produced the best amplification (Fig. 1A), evidenced by steeper slopes during the exponential phase and higher fluorescence plateaus. This corresponded to lower Ct values at the 2× buffer concentration for both higher (10⁶) and lower (10²) ecRNA copy numbers (Fig. 1B). For the dataset from the 10^6^ copy number, a one-way repeated measures ANOVA (RM ANOVA) revealed a significant effect of buffer condition on amplification efficiency (*F*(4,12) = 3.48, *P* = 0.042). However, post-hoc Tukey comparisons could not identify any specific group differences. For the dataset from the 10² copy number, a RM ANOVA also revealed a significant effect of buffer condition on amplification efficiency (*F*(4, 11) = 53.55, *P* < 0.001). In addition, the Post-hoc Tukey comparisons showed that the 1×, 1.5×, and 2.5× Platinum II Taq buffer reactions produced significantly higher (all *P* < 0.001) amplification than both the RNA Ultrasense and 2× Platinum II conditions (*P* < 0.001).

***Optimal reverse transcriptase: Taq polymerase ratio***. Reverse transcriptase is a known inhibitor of Taq polymerase [[20], [21], [22], [23], [24]]. To address this, we tested various concentrations of Superscript IV reverse transcriptase combined with 1 U of the Platinum II *Taq* polymerase. At high input (10⁶ copies/well; Ct ≈ 20) amplification curves were similar across reverse transcriptase concentrations, except at 0.1 U of Superscript IV, where the Ct value was slightly, but significantly higher (*P* < 0.05) compared to RNA Ultrasense. This reflects the abundant enzyme activity early in cycling (Fig. 2A). At 10^2^ ecRNA copy numbers per well, Ct values were similar across SuperScript IV concentrations 0.2 U to 1.6 U, being significantly lower (*P* < 0.05) than RNA Ultrasense (Fig. 2B). The amplification plots reveal an inhibitory effect at higher RT levels. At lower ecRNA copy numbers per well (10^3^ and 10^2^), SuperScript IV concentrations of 0.4–1.6 U per well progressively displayed shallower slopes during the exponential phase with lower fluorescence plateaus, consistent with dose-dependent inhibition of the Taq polymerase (Fig. 2A). This effect likely arises because late occurring amplification is more susceptible to limiting polymerase activity and RT interference. Based on the amplification plots, 0.2 U of the SSIV appeared to be the best ratio to use with 1 unit of the Platinum II *Taq* polymerase, which we henceforth defined as 1× enzyme mix.

**Fig. 3:**
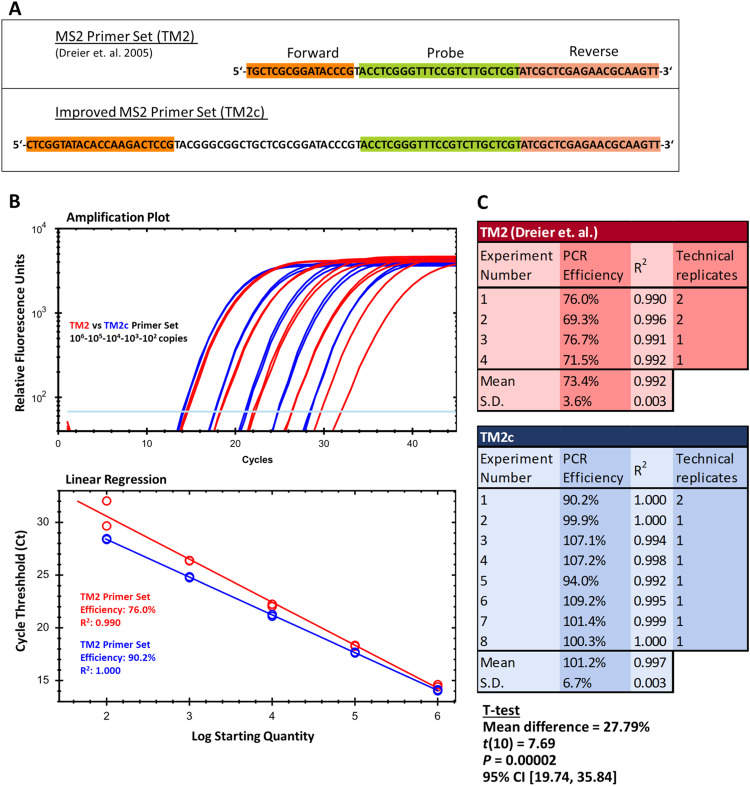
Optimization of PCR primers against process control virus MS2. (A) Primer-probe placement on the amplified sequence of the MS2 genome. (B) Comparison of representative amplification plots and process-control RNA standard-curve regressions for MS2 using Dreier et al. primers (red) and the optimized TM2c forward primer (blue), as per ISO 15216-2:2019 standard-curve construction. (C) Reproducibility of MS2 PCR efficiency for the original (TM2) and optimized (TM2c) primer sets across independent one-step RT-qPCR experiments.

***Optimized MS2 detection primer.*** MS2 is an easily cultivable RNA virus (bacteriophage) whose host is the *Escherichia coli* bacterium. In the process of establishing the detection of the process control virus MS2, it was found that the TM2 primers proposed by Dreier et al [1] yielded consistently poor amplification efficiencies of <90 % with purified MS2 RNA (Fig. 3B). Looking at the primers in detail, we found that the probe was placed almost directly next to the forward primer, with just 1 base pair between them (Fig. 3A). This leaves little space for the polymerase to potentially interact with the DNA matrix. Suitable primer binding sites upstream of the original forward primer sequence were located with Primer Blast [14]. With the improved forward primer (TM2c), amplification efficiency improved to above 90 %. Performance of the new primer set gave consistently good amplification efficiencies between 90–110 % with various one-step RT-qPCR mixes (Fig. 3C). An independent two-sample *t*-test showed that PCR efficiencies obtained using the TM2c primer set were significantly higher than that from the original TM2 primer set (mean difference = 27.79 %; *t*(10) = 7.69, *P* = 0.00002; 95 % CI [19.74, 35.84]).

**Fig. 4:**
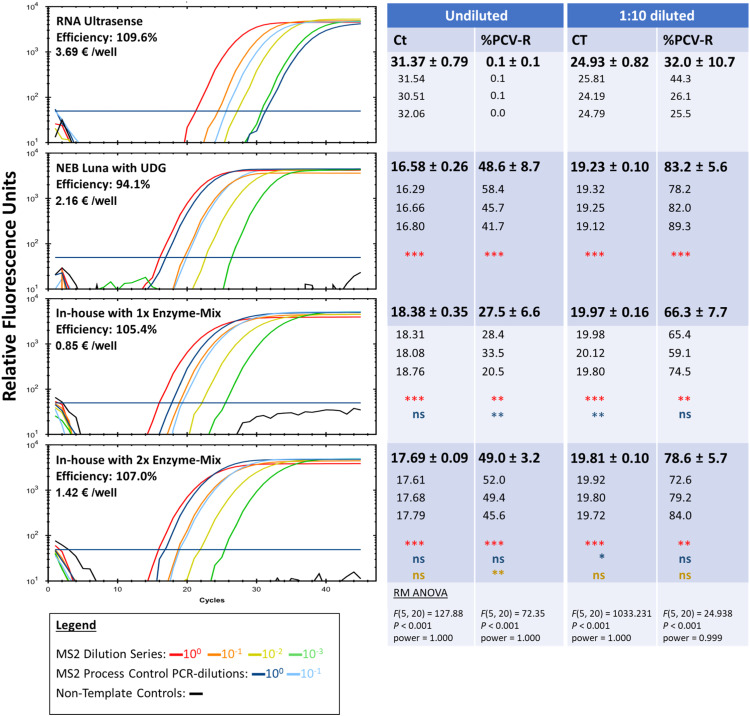
PCR efficiency and recovery of the process control virus MS2 from raspberries using two commercial one-step RT-qPCR kits and the in-house mix using 1× vs 2× enzyme concentrations. Left panel: representative amplification plots from 4 different mixes. Price per well was calculated based on 25 µL reaction volume and on manufacturer pricing before taxes as of April 2025 in Germany. Right panel: Mean (bold) Ct and MS2 process control virus recovery ( %PCV-R) ± SD for three independent raspberry samples (n = 3). Individual sample values are shown beneath each mean. Results representative of 2 independent experiments. Statistical analysis: repeated-measures one-way ANOVA (RM-ANOVA) with Tukey’s post hoc test; red symbols denote comparisons to RNA UltraSense, blue symbols denote comparisons to NEB Luna with UDG and brown symbols denote comparisons to 1x enzyme-mix. Significance levels: **P < 0.01, ***P < 0.001; not significant (ns).

***Comparison to commercial one-step RT-qPCR kits***. Next, we evaluated the performance of the in-house, one-step RT-qPCR mix, using both 1× and 2× enzyme concentrations, in comparison to two commercial one-step RT-qPCR kits. The recovery rate of the process control virus MS2 from raspberries was used as an indicator of inhibitor resistance. In undiluted RNA samples, the presence of co-purified PCR inhibitors in the viral RNA compromised the performance of the RNA UltraSense kit, resulting in poor detection of undiluted process control virus RNA, characterized by significantly lower (*P* < 0.01) virus recovery (Fig. 4). Although 2 independent experiments were performed for the comparison, the RNA UltraSense Kit had a poor PCR efficiency in the second experiment (Supplementary Data). At 1:10 RNA dilution however, the recovery rate aligned more with other tested mixes, although it was still significantly lower (*P* < 0.01). At both dilutions over both experiments, the Ct values obtained from the in-house mixes and the NEB Luna UDG mix were substantially lower (*P* < 0.001) than that obtained from the RNA UltraSense kit (Fig. 4, Supplementary Data). This highlights the critical role of a one-step RT-qPCR mix with strong resistance to PCR inhibitors in achieving successful virus detection. Both the 1× and 2× in-house enzyme mixes outperformed the RNA Ultrasense kit, with the 2× mix showing slightly better virus recovery than the 1x mix un undiluted RNA samples (*P* < 0.01)and comparable performance to the NEB Luna Probe One-Step RT-qPCR kit with UDG (Fig. 4). Henceforth, the 2× enzyme mix was used for one-step RT-qPCR on berry samples and the final composition is given in Table 2.

**Table 2:** Final composition of the in-house, one-step RT-qPCR Mix.

Reagent	Source	Stock solution	Volume (µL) per reaction	Price per reaction (€)▲
SuperScript IV RT	Thermo Fisher	See ◊	0.4000	0.02
Platinum II Taq HS				1.14
Platinum II Taq PCR Buffer	Thermo Fisher	5x	10.0000	NA
Dithiothreitol	PanReac Applichem	100 mM	0.1250	Neg
dNTP	NEB	10 mM each	1.0000	0.07
UltraPure BSA	Thermo Fisher	50 mg/mL	0.4000	0.09
Primers-probe-mix	NA	See*	0.4125	NA
RNase Inhibitor Hu	Qiagen	40 U/µL	0.1250	0.10
Template RNA	NA	NA	5.0000	NA
Total volume (make up with nuclease free water)			25.0000	1.42

▲ based on manufacturer pricing from April 2025 without VAT.

◊ 1 µL of the SuperScript™ IV Reverse Transcriptase (RT) enzyme (200 U/µL) was added to 200 µL of the Platinum™ II Taq Hot-Start (HS) DNA Polymerase enzyme (5 U/µL) to make the enzyme stock solution.

⁎ The primers-probe-mix stock solution for each virus detection was put together as follows: 30.3 µM forward primer, 54.55 µM reverse primer, 15.15 µM probe. In a multiplex detection, multiple primers-probe-mix for each virus was added to the reaction. NA Not applicable Neg Negligible.

**Fig. 5:**
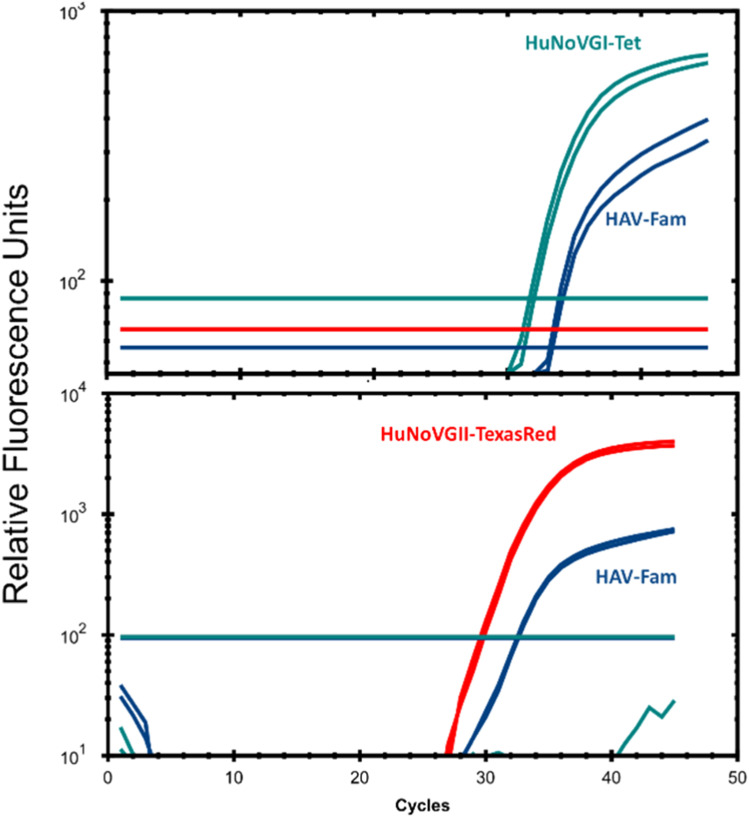
Multiplex detection of viruses in 2 samples of strawberries contaminated with HuNoV-GI and HAV (upper panel) or HuNov-GII and HAV (lower panel) using the in-house, one-step RT-qPCR mix.

***Multiplex detection of viruses in strawberries with in-house, one-step RT-qPCR mix.*** Other labs have reported that multiplex virus detection is feasible, albeit with slightly lower detection limits [[25], [26], [27]] and we successfully used our in-house mix for multiplex virus detection. Strawberry samples were artificially contaminated as part of a comparative laboratory study organized by the National Reference Laboratory for Foodborne Viruses. Using the in-house, one-step RT-qPCR mix with 2× enzyme concentration, 2 strawberry samples each contaminated with two different viruses (HAV + HuNoV-GI and HAV + HuNoV-GII) were successfully purified and detected using a multiplex approach (Fig. 5). Our results thus appear to indicate the suitability of our in-house mix for use in multiplex detections.

## Limitations

Although previous studies have described in-house kits based on enzyme purification [[28], [29], [30]], we relied exclusively on commercially available enzymes to ensure time efficiency, consistent quality, and reliability. Fully home-purified systems, however, may offer greater cost savings but are limited to laboratories equipped for protein expression and purification, as well as protein standardization and quality control. Likewise, other cost-effective commercial enzyme alternatives likely exist, though they too would require careful optimization of reaction buffer, reverse transcriptase and Taq polymerase concentrations to avoid inhibition while maintaining sufficient cDNA yield.

We were able to reduce per-sample costs from €148 (RNA Ultrasense) to €57 (in-house mix), with a further reduction to 26€ in a multiplex setting (prices from Germany, April 2025; before VAT). The NEB Luna UDG master mix performed equivalently to the in-house mix, but at a higher cost. Nevertheless, initial reagent procurement and primer synthesis might still represent a barrier for some low-resource laboratories. Future work should also validate this protocol across more diverse food matrices and control viruses to ensure universal reproducibility. Additional savings could be achieved by lowering enzyme concentrations for matrices with minimal PCR inhibition, but this must be empirically tested for each matrix–enzyme combination.

## Ethics statements

None

## Supplementary material *and/or* additional information [OPTIONAL]

Supplementary Table 1. Gene synthesis sequences and amplification primers for PCR-based production of ecRNA.

The raw data for the figures and the results of the statistical analysis are provided in the Excel file "mmc1.xlsb".

## Declaration of generative AI and AI-assisted technologies in the writing process

During the preparation of this work the authors used OpenAI’s ChatGPT (version 4o-mini) in order to improve clarity in select sections of the text. After using this tool, the authors reviewed and edited the content as needed and take full responsibility for the content of the publication.

## CRediT authorship contribution statement

**Hui Zhi Low:** Writing – original draft, Writing – review & editing, Conceptualization, Methodology, Investigation, Visualization, Funding acquisition. **Christina Böhnlein:** Writing – review & editing, Resources, Conceptualization, Funding acquisition. **Charles M.A.P. Franz:** Writing – review & editing, Resources, Supervision, Funding acquisition.

## Declaration of competing interest

The authors declare that they have no known competing financial interests or personal relationships that could have appeared to influence the work reported in this paper.

## Data Availability

Data will be made available on request.

## Appendix Supplementary materials

Image, application 1

Image, application 2
