# Supplementary material for: Development of an in-house, one-step RT-qPCR mix and optimized MS2 detection primers for hepatitis A virus and norovirus detection in berries
Source: MethodsX. 2025 Nov 1;15:103703. doi: 10.1016/j.mex.2025.103703 (PMC12648481; doi:10.1016/j.mex.2025.103703)
Supplement: Supplementary file 2 [file mmc2.docx]

Supplementary Table 1

| Target | Sequence (5’-3’) | Amplification Primers (5’-3’) | Remarks |
| --- | --- | --- | --- |
| HAV | GTTGTAGTTCTCCGGCGTGTAATACGACTCACTATAGTCACCGCCGTTTGCCTAGGCTATAGGCTAAATTTTCCCTTTCGGATCCCCCTTTCCTATTCCCTTTGTTTTGCTTGTAAATATTGATTTGTAAATATTGATTCCTGCAGGTTCAGGGTTCTTAAATCTGTTTCTCTATAAGAACACTCATTTCACGCTTTCTGTCTTCTTTCTTCCAGGGCTCTCCCTAGAATTACCTACCGGCGT | Forward:  GTTGTAGTTCTCCGGCGTG  Reverse:  ACGCCGGTAGGTAATTCTAG | Synthesized as GeneStrands (Eurofins) |
| HuNoV-GI | CCACCATACCTTCGATATTCTAATACGACTCACTATAGCGCTGGATGCGCTTCCATGACCTCGGATTGTGGACAGGAGATCGCGATCTTCTGCGGATCCGAATTCGTAAATGATGATGGCGTCTAAGGCGATTGCCCGCTAAGATATT | Forward:  CCACCATACCTTCGATATTC  Reverse:  AATATCTTAGCGGGCAATCG | Synthesized as GeneStrands (Eurofins) |
| HuNoV-GII | AGCAGGTATAAGGTCGTCCTAATACGACTCACTATAGATGTTCAGATGGATGAGATTCTCAGATCTGAGCACGTGGGAGGGCGATCGCAATCTGGCTCGGATCCCCAGCTTTGTGAATGAAGATGGCGTCGACAGCACTTCAGCCAAGGAA | Forward:  AGCAGGTATAAGGTCGTCC  Reverse:  TTCCTTGGCTGAAGTGCTG | Synthesized using standard gene synthesis (Eurofins) |
| Legend:  Random 20mer forward primer binding site  Random 20mer reverse primer binding site  T7 Promoter | | | |
